# Course of SP-D, YKL-40, CCL18 and CA 15-3 in adult patients hospitalised with community-acquired pneumonia and their association with disease severity and aetiology: A post-hoc analysis

**DOI:** 10.1371/journal.pone.0190575

**Published:** 2018-01-11

**Authors:** Simone M. C. Spoorenberg, Stefan M. T. Vestjens, G. P. Voorn, Coline H. M. van Moorsel, Bob Meek, Pieter Zanen, Ger T. Rijkers, Willem Jan W. Bos, Jan C. Grutters, the Ovidius study group

**Affiliations:** 1 Department of Internal Medicine, St Antonius Hospital, Nieuwegein, The Netherlands; 2 Department of Medical Microbiology and Immunology, St Antonius Hospital, Nieuwegein, The Netherlands; 3 Department of Pulmonology, St Antonius Hospital, Nieuwegein, The Netherlands; 4 Division of Heart and Lungs, University Medical Centre Utrecht, Utrecht, The Netherlands; 5 Department of Sciences, Roosevelt Academy, Middelburg, The Netherlands; Universita degli Studi di Pavia, ITALY

## Abstract

**Background and aim:**

SP-D, YKL-40, CCL18 and CA 15–3 are pulmonary markers that have been extensively investigated in different chronic pulmonary diseases. However, in acute pulmonary diseases, such as community-acquired pneumonia (CAP), little is known about the course of these markers and their relationship with the aetiological agent. The aim of this study was to investigate the course of these four markers in CAP and to study influence of disease severity, aetiology and antibiotic use prior to admission on their course.

**Methods:**

We included 291 adult patients hospitalised with CAP and 20 healthy controls. Measurements were performed in serum of day 0, 2, and 4, and at least 30 days after admission.

**Results:**

Our most important results were: 1) At all time-points, including 30 days after admission, YKL-40 and CCL18 levels were higher in CAP patients compared to healthy controls; and 2) Patients with CAP caused by an intracellular, atypical bacterium had lower YKL-40 and especially CCL18 levels on and during admission in comparison with other or unknown CAP aetiology.

**Conclusions:**

Our findings suggest that these pulmonary markers could be useful to assess CAP severity and, especially YKL-40 and CCL18 by helping predict CAP caused by atypical pathogens.

## Introduction

The markers surfactant protein-D (SP-D), YKL-40, chemokine (C-C motif) ligand (CCL)18 and cancer antigen (CA) 15–3 have been extensively investigated in different chronic pulmonary diseases. Blood levels of all these four, mainly pulmonary, markers, are predictors of disease severity and or prognosis in idiopathic pulmonary fibrosis[1–5] and of pulmonary involvement in systemic sclerosis[6–9]. Furthermore, SP-D, CCL18 and YKL-40 levels have been associated with chronic obstructive pulmonary disease (COPD) severity.[10–12]. Studies focusing on the value of these markers in acute pulmonary disease, like community-acquired pneumonia, (CAP) are scarce. Recently, we demonstrated that levels of YKL-40, CCL18 and SP-D on hospital admission are associated with disease severity and mortality in CAP.[13] The course of these markers and their relationship with aetiology in CAP is unknown. SP-D is a collagenous C-type lectin synthesised by lung epithelial cells. It decreases surface tension at the air-blood interface and plays a role in pulmonary host defence.[14,15] YKL-40 or human cartilage glycoprotein-39 is a matrix protein in specific granules of human neutrophils and most likely has a role in the inflammatory process by facilitating cell migration through extracellular matrix and by tissue remodelling after an inflammatory response. [16, 17] Compared to healthy controls, YKL-40 levels are higher in patients hospitalised with CAP.[13,18] CCL18, or macrophage inflammatory protein 4, is a cytokine involved in attracting naive T-cells, T-regulatory cells, T-helper 2 cells, dendritic cells, basophiles and B-cells and is produced by dendritic cells, monocytes and macrophages.[19–21] CCL18 has a role in tissue repair and is induced by fibroblasts through an Sp1-dependent pathway that required basal Smad3 activity.[22, 23] CA 15–3, also known as mucin-1 and Krebs von de Lungen-6, is a glycoprotein produced by secretory epithelia, including lung epithelial cells, forming a protective layer. In addition, it is involved in cell-cell communication.[24] CA 15–3 level elevation has been associated with interstitial lung disease. [25] However, CA 15–3 is best known for its use as a diagnostic biomarker in breast cancer. To the best of our knowledge, both CCL18 and CA 15–3 haven’t been investigated in CAP.

CAP is a disease with high morbidity and mortality rates. To improve outcomes of CAP, better understanding of pathophysiological mechanisms might help to improve current therapies and detect new treatment options. We assessed the course of SP-D, CA 15–3, CCL18 and YKL-40 in patients hospitalised with CAP and the influence of disease severity, causative pathogens, antibiotic use prior to admission, and use of dexamethasone on their course.

## Materials and methods

### Patients

Adult patients hospitalised with CAP who participated in a randomised controlled trial (NCT00471640), conducted between November 2007 and September 2010 in the St. Antonius Hospital in Nieuwegein and the Gelderse Vallei Hospital in Ede, were enrolled.[26] Patients aged 18 years or older were included in case of a confirmed community-acquired pneumonia which consisted of a new pulmonary infiltrate on the chest radiograph in combination with two or more of the following criteria: sputum production, cough, temperature >38°C of <35°C, auscultatory findings consistent with pneumonia, CRP >15 mg/L, white blood cell count >10x10^9^ cells/L or <4x10^9^ cells/L, or >10% of rods in leucocyte differentiation. Exclusion criteria contained known congenital or acquired immunodeficiency, chemotherapy, use of corticosteroids or other immunosuppressive medication in the previous six weeks, haematological malignant disease, necessity of corticosteroids treatment of immediate admission on the intensive care unit, and pregnant or breastfeeding women. All patients provided written informed consent. This trial evaluated the effect of a four day course of adjunctive intravenous dexamethasone in 304 patients; a reduction of one day in length of hospital stay in the dexamethasone group was found.[26] Extensive microbiological testing was performed, including detection for atypical pathogens by TaqMan real-time polymerase chain reactions (PCRs) and paired complement fixation test (CFT). For CFT, a fourfold or greater increase of antibody titre in serum from at least ten days with a maximum of 120 days after admission compared to the titre in serum of day of admission was considered indicative for an acute infection. Further details of the microbiological investigations can be found in S1 File. The study was approved by the Medical Ethical Committees of the St. Antonius Hospital and the Gelderse Vallei Hospital.

A control group comprised 20 healthy, non-smoking volunteers selected by age and gender: 11 males and 9 females, with a mean age of 60.9 years (SD 3.7).

### Measurement of markers

Blood was sampled and stored on day of admission before randomisation (and therefore before the first dose of study medication was given, hereafter called ‘day 0’), if still admitted on days 2 and 4, and during an outpatient visit at least 30 days after admission (hereafter called ‘day 30’). If no serum sample was available from day 2, serum of day 1 (when present) was used instead. In case no day 4 serum sample was stored, serum of respectively days 3 or 5 were used if available. Of 291/304 (95.7%) patients sample were available for measurement of pulmonary markers; 145 patients who received dexamethasone and 146 patients who received placebo. S1 Table shows differences in baseline characteristics between patients of whom serum of day 4 and day 30 were available, compared to patients of whom these samples were not available for measurement of pulmonary markers.

YKL-40, CCL18, SP-D and CA 15–3 levels were determined simultaneously by two separate duplex bead-based immunoassay (R&D Systems) in accordance with the manufacturer’s instructions. Pulmonary marker levels were measured on a Bio-Plex System (Bio-Rad). Details of quantification levels can be found in S1 File. Pulmonary markers were compared to interleukin-6 (IL-6) and C-reactive protein (CRP). CRP was prospectively measured with high sensitive-CRP (Roche Diagnostics GmbH, Mannheim, Germany) during admission. IL-6 levels were measured in batch by Milliplex multianalyte profiling (Millipore, Billerica, MA, USA).

### Data analyses

Variables were stated as number with percentage, mean with standard deviation (SD) or median with interquartile range [IQR]. Differences in marker levels between patients and healthy controls were calculated using Mann-Whitney U test. The six markers all proved to be naturally log-normally (ln) distributed, hence we ln-transformed values prior to following analysis. To evaluate levels per pathogen, pathogens were categorised into four groups: group 1 contained all extracellular bacteria including *Streptococcus pneumoniae*, group 2 contained the intracellular, atypical bacteria (*Coxiella burnetii*, *Chlamydia* species, *Legionella* species and *Mycoplasma pneumoniae*), group 3 consisted of viruses, and group 4 of unknown aetiology. Possible differences in baseline characteristics between these four aetiological groups were calculated with the Kruskal-Wallis test or one-way ANOVA, where appropriate. For this analysis, a *p*-value<0.0024 was considered significant (Bonferroni correction applied).

As the primary aim of the study relates to the change of the markers over time, data were analysed with repeated measures linear mixed modelling with random intercepts / slopes with a first order auto-regression covariance matrix.[27] Variables that possibly influenced course of the markers were used in model building: the repeated observations (hereafter called ‘time’), pneumonia severity index (PSI) classes 4–5, aetiology, antibiotic use before hospitalisation, dexamethasone use, COPD-presence and the interaction of each variable with time. Further details on this analysis can be found in S1 File.

Finally, receiver operating characteristics (ROC) and regression analyses were used to evaluate the predictive value of a given pulmonary marker level on admission for atypical CAP aetiology, using intracellular bacteria versus all other aetiology (extracellular bacteria, viruses and unknown). Further details of these analyses can be found in S1 File.

Power analysis of the original trial was based on length of stay. Data were analysed with SPSS statistical software for Windows version 22.0. Figures were drawn with GraphPad Prism 6 for Windows, version 6.01. For all analyses except when a Bonferroni correction was applied, a *p*-value of <0.05 was considered statistically significant.

## Results

### Patients

Of 291/304 (95.7%) patients, samples were available for measurement of pulmonary markers. S2 Table shows the available levels of YKL-40, CA 15–3, CCL18, SP-D, CRP, and IL-6 for these 291 patients. Baseline characteristics and outcomes of the 291 patients can be found in Table 1. Atypical pathogens were detected in 55 patients; in 12 patients by serology, in eight patients by PCR, in 21 by both serology and PCR and the remaining by urine antigen test or blood or sputum culture. Viral pathogens were detected as CAP causing pathogen in 19 patients and mainly concerned Influenza virus (seven patients), three respiratory syncytial virus, and three with para-influenza virus.

**Table 1:** Baseline characteristics and outcome of 291 patients hospitalised with community-acquired pneumonia, divided in four aetiological groups.

	All patients (n = 291)	Intracellular bacteria (n = 55)	Extracellular bacteria (n = 90)	Viruses (n = 19)	Unknown (n = 127)
Male sex (%)	163 (56.0)	37 (67.3)	41 (45.6)	15 (78.9)	70 (55.1)
Age (year) (±, IQR)	64.0 (18.5, 52.0–79.0)	54.9 (15.8, 44.0–69.0)	61.7 (19.0, 50.8–76.3)	70.5 (19.5, 60.0–82.0)	68.6 (17.4, 17–93)*
*Comorbidities (%)*					
Chronic kidney disease	28 (9.6)	3 (5.5)	9 (10.0)	3 (15.8)	13 (10.2)
Diabetes mellitus	41 (14.1)	4 (7.3)	20 (22.2)	2 (10.5)	15 (11.8)
Liver disease	2 (0.7)	0	1 (1.1)	0	1 (0.8)
Neoplastic disease	19 (6.5)	3 (5.5)	7 (7.8)	0	9 (7.1)
Chronic heart failure	47 (16.2)	7 (12.7)	10 (11.1)	3 (15.8)	27 (21.3)
COPD	32 (11.0)	3 (5.5)	13 (14.4)	1 (5.3)	15 (11.8)
*PSI classes (%)*					
Classes 1–3	152 (52.2)	34 (61.8)	46 (51.1)	10 (52.6)	62 (48.8)
Classes 4–5	139 (47.8)	21 (38.2)	44 (48.9)	9 (47.4)	65 (51.2)
Systolic blood pressure in mm Hg (±)	132 (22)	131 (14)	128 (23)	131 (26)	134 (23)
Temperature in °C (±)	38.2 (1.1)	38.4 (1.1)	38.2 (1.0)	38.1 (1.0)	38.1 (1.2)
*Laboratory parameters*:					
CRP in mg/L [IQR]	214 (96–329)	288 (203–347)	299 (136–388)	196 (130–277)	129 (58–249)*
WBC in 10^9^/L (±)	14.4 (6.5)	10.4 (3.9)	17.0 (6.6)	14.0 (6.3)	14.3 (6.5)*
Procalcitonin in ug/L [IQR]^†^	0.5 (0.2–3.1)	0.4 (0.2–2.0)	2.4 (0.4–8.3)	0.4 (0.2–1.3)	0.3 (0.1–1.5)*
Interleukin-6 [IQR]	45.6 (14.0–194.0)	39.6 (15.9–86.7)	183.5 (35.3–794.2)	17.3 (7.9–71.3)	29.7 (11.8–114.6)*
YKL-40 in ng/mL [IQR]	189 (67–455)	70 (33–196)	331 (112–740)	282 (125–650)	182 (67–373)*
CCL18 in ng/mL [IQR]	77 (43–125)	38 (26–57)	98 (69–152)	80 (38–134)	81 (51–129)*
SP-D in ng/mL [IQR]	6.3 (3.0–11.0)	5.9 (2.3–8.7)	6.3 (3.1–11.1)	3.9 (2.4–9.5)	7.8 (3.4–11.9)
CA 15–3 in U/mL [IQR]	23.8 (14.0–37.5)	22.4 (12.6–35.9)	21.6 (12.4–32.1)	22.4 (12.2–43.9)	28.5 (15.9–43.1)
Days ill before admission [IQR]	4.5 (3–7)	5.0 (4.0–6.0)	4.0 (2.0–7.0)	4.0 (2.0–7.0)	5.0 (3.0–7.0)
Antibiotics before hospitalisation	80 (27.5)	19 (34.5)	24 (26.7)	5 (26.3)	32 (25.2)
Dexamethasone (%) ^‡^	145 (49.8)	26 (47.3)	47 (52.2)	5 (26.3)	67 (52.8)
*Outcomes*					
Length of hospital stay [IQR]	7.0 (5.0–10.5)	5.5 (3.5–8.5)	7.5 (5.5–10.8)	7.5 (5.5–9.0)	7.0 (5.0–10.5)
ICU admission (%)	16 (5.5)	3 (5.5)	8 (8.9)	0	5 (3.9)
30-days mortality (%)	19 (6.5)	2 (3.6)	4 (4.4)	2 (10.5)	10 (7.9)

Data are shown as number (%), mean (standard deviation) or median [interquartile range].

* Indicates a significant result after Bonferroni correction for multiple testing, a *p*-value<0.0023

^‡^ A four day dexamethasone course was given as part of a randomised clinical trial

^†^ Of 16 patients data were missing.

Significance is calculated using Kruskal-Wallis or One-way ANOVA, as appropriate.

Abbreviations: COPD, chronic obstructive pulmonary disease; CRP, C-reactive protein; PSI, pneumonia severity index; ICU, intensive care unit; IQR, interquartile range; *S*. *pneumoniae*, *Streptococcus pneumoniae*; WBC, white blood cell count.

### Pulmonary marker levels in CAP and in healthy controls

Median levels of the pulmonary markers in patients and healthy controls are shown in Table 2. On all four time-points YKL-40 and CCL18 levels were significantly higher in patients with CAP compared to healthy controls. For CA 15–3 and SP-D, differences between patients and controls were smaller.

**Table 2:** Serial levels of the four pulmonary markers in 291 patients hospitalised with community-acquired pneumonia and in 20 healthy controls.

	Day 0	Day 2	Day 4	Day 30	Healthy controls
YKL-40	189 (67–456)*	99 (45–227)*	87 (43–182)*	44 (26–99)*	23 (13–37)
CCL18	77 (43–123)*	73 (40–114)*	77 (51–116)*	63 (47–89)*	38 (28–51)
SP-D	6.3 (3.0–11.0)*	11.8 (7.4–18.5)	15.2 (10.0–22.7)*	9.7 (6.7–15.4)	9.8 (5.9–14.3)
CA 15–3	23.8 (14.0–37.5)	23.7 (13.3–38.7)	26.0 (14.2–42.9)*	27.4 (16.9–44.5)*	19.5 (11.8–25.7)
C-reactive protein	214 (96–329)	118 (64–227)	52 (28–120)	5 (5–10)	
Interleukin-6	47.2 (15.7–198.0)	11.6 (3.3–28.2)	9.9 (3.6–29.5)	3.9 (0.5–13.1)	

Median with interquartile ranges are shown.

* Indicates a significant difference (*p*-value <0.05) compared to the healthy control group, calculated using Mann-Whitney U test.

### Mixed model analysis

Table 1 shows differences in baseline between the four aetiological groups. YKL-40, CCL18, CA 15–3, CRP and IL-6 differed significantly on admission between the aetiologies, as did age, white blood cell count, and procalcitonin. S3 Table shows which variables were added to the final linear mixed model for each investigated marker. Hereafter, the effect of each variable on marker course will be discussed separately.

#### PSI score (see “Fig 1”)

**Fig 1:**
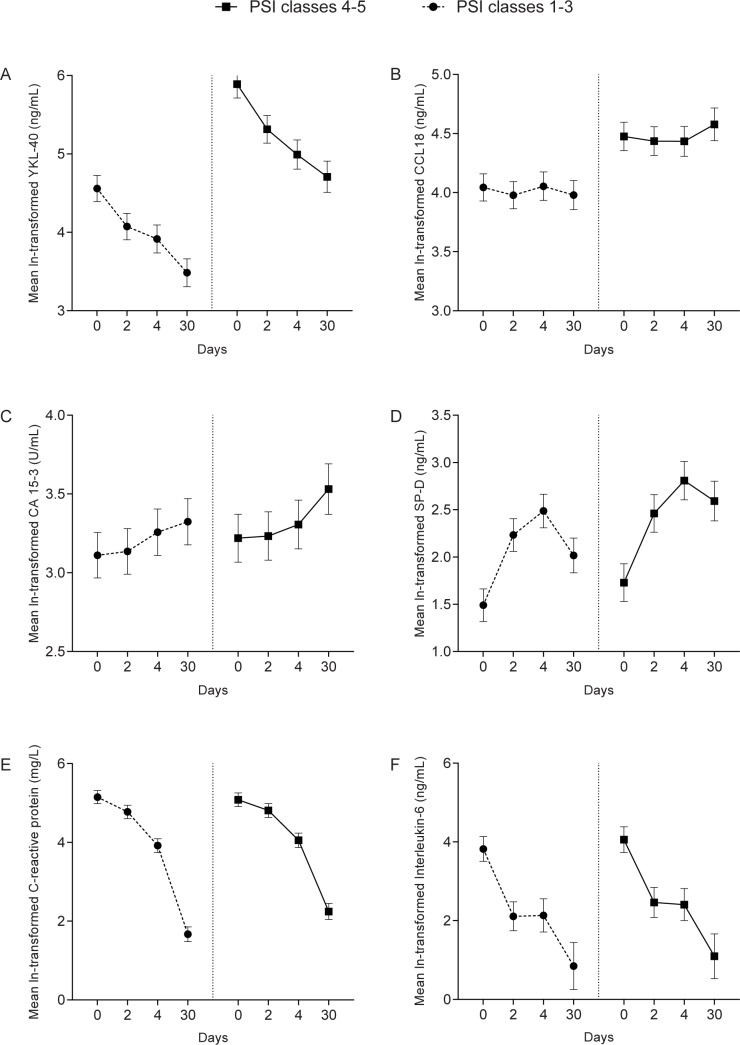
Change in log normally-transformed pulmonary marker levels over time for pneumonia severity index score. Data are shown as estimated marginal means with 95% confidence intervals.

Mean YKL-40, CCL18, SP-D and to a lesser extent CRP levels were significantly higher in patients in PSI classes 4–5, compared with those in PSI classes 1–3. The difference in YKL-40 was not time dependent: even on day 30 YKL-40 levels were still higher. In contrast, CCL18 and SP-D differences were time dependent (*p*:0.017 and *p*:0.014, respectively): on day 4 CCL18 mean level was exp^0.380^ (= 1.46, 95% CI 1.45–1.47) times higher in PSI classes 4–5 which increased to exp^0.598^ (= 1.82, 95% CI 1.79–1.85) on day 30 (see “Fig 1B”). The largest SP-D difference was also seen on day 30, when mean level was exp^0.575^ (= 1.78, 95% CI 1.73–1.83) times higher in PSI classes 4–5 (see “Fig 1D”). IL-6 levels did not differ between PSI severity groups.

#### Aetiology (see “Fig 2”)

**Fig 2:**
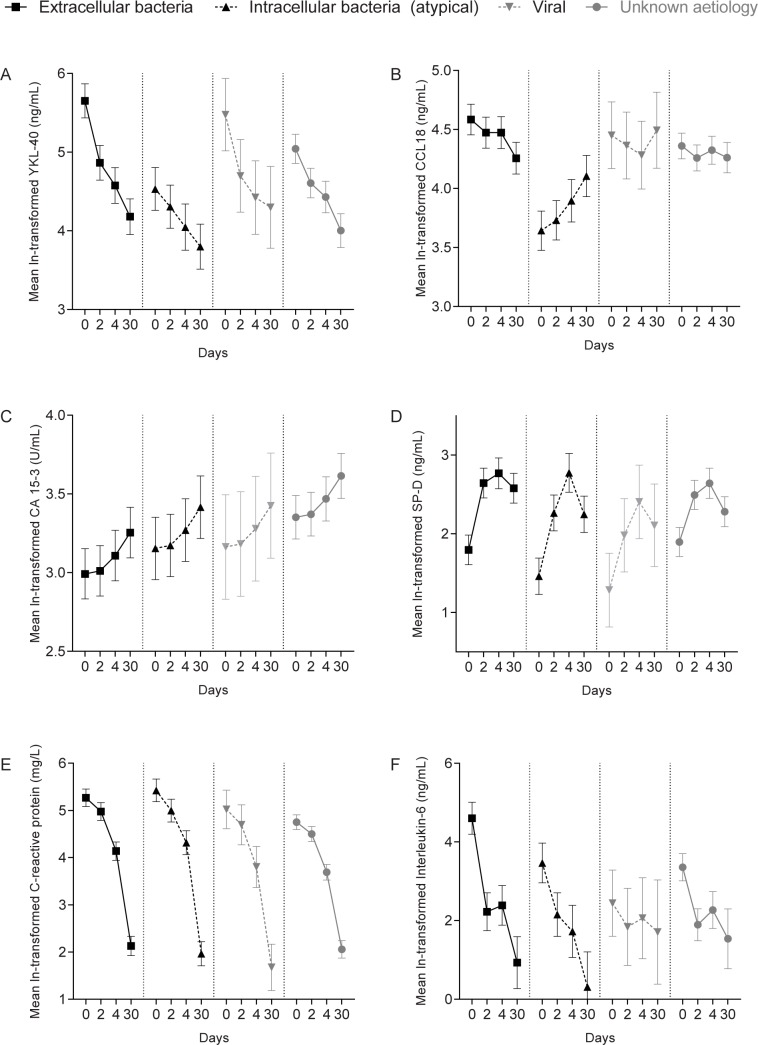
Change in log normally-transformed pulmonary marker levels over time for each aetiological group. Data are shown as estimated marginal means with 95% confidence intervals.

Mean YKL-40 and CCL18 levels of the intracellular, atypical pathogens group were significantly lower compared to the extracellular bacteria group (both *p*<0.001), the viruses (*p*:0.019 and *p*<0.001, respectively) and unknown aetiology group (*p*:0.016 and *p*<0.001, respectively). In turn, YKL-40 levels in patients with extracellular bacteria were significantly higher compared to the unknown group (*p*:0.015); for CCL18, a trend was seen (*p*:0.053). For both markers, the differences were dependent on time (both p<0.001). Especially on day 0, YKL-40 levels differed between the groups, with mean levels of exp^4.531^ (= 92.9), exp^5.042^ (= 154.8), exp^5.476^ (= 238.9) and exp^5.652^ (= 284.9) ng/mL for intracellular, viral, unknown, and extracellular pathogen respectively (see “Fig 2A”). For CCL18, mean levels were lower in the intracellular bacteria group on days 0, 2 and 4, while levels on day 30 were comparable to the other two aetiologies (see “Fig 2B”).

For CA 15–3, the extracellular bacteria group had lower mean levels compared to the unknown group (*p*<0.001)(see “Fig 2C”). For SP-D, patients with a viral pneumonia had lower levels compared to the extracellular bacteria group (*p*:0.022) and compared to the unknown group a trend was seen (*p*:0.078) (see “Fig 2D”). CRP levels were lowest in the unknown group (both *p*<0.001 compared to intra- and extracellular bacteria) and in the viruses (*p*:0.041 and *p*:0.061 compared to intra- and extracellular bacteria, respectively). IL-6 levels did not differ significantly between the four aetiological groups, but levels did change over time.

#### Antibiotic use at home (see S1 Fig)

In patients who received antibiotic treatment prior to hospital admission, significantly lower levels of YKL-40, CA 15–3 and IL-6 were found, compared to patients that were not pre-treated. Trajectories of CCL18, SP-D and CRP levels were not affected by antibiotic use before.

#### Dexamethasone (see S2 Fig)

CCL18 mean levels were lower in the dexamethasone group compared to levels in the placebo group. This difference was time dependent (*p*<0.001): on days 2 and 4 mean levels were respectively exp^0.401^ (= 1.49, 95% CI 1.48–1.51) and exp^0.537^ (= 1.71, 95% CI 1.70–1.73) times lower in the dexamethasone group. For SP-D, mean levels significantly changed over time due to dexamethasone treatment (*p*:0.023): on day 2 and 4 levels were slightly higher in the dexamethasone group (exp^0.148^ (= 1.16, 95% CI 1.13–1.19) and exp^0.119^ (= 1.13, 95% CI 1.09–1.16) times respectively, see S2 Fig). CRP levels and IL-6 levels were also reduced by dexamethasone. Dexamethasone treatment had no effect on YKL-40 levels or CA 15–3 levels.

### Predictive value for intracellular (atypical) pathogens

**Fig 3:**
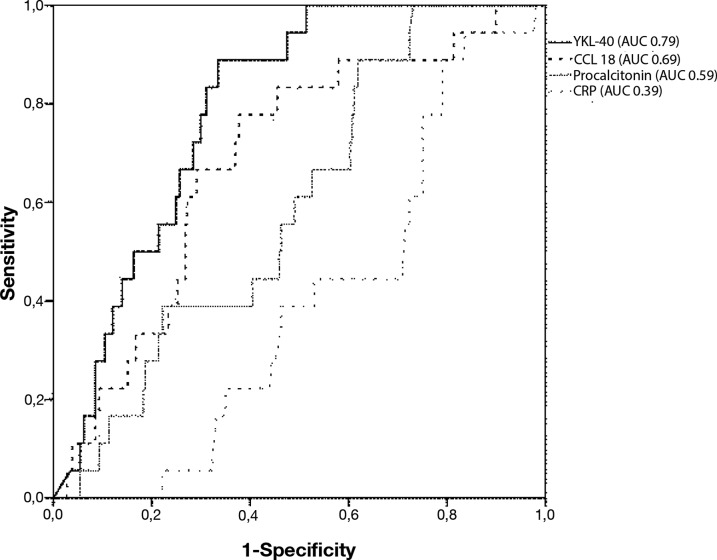
Area under the curve for YKL-40, CCL18, CRP and procalcitonin levels on admission for intracellular aetiology of community-acquired pneumonia.

Since YKL-40 and CCL18 levels differed most between the intracellular bacteria group and the other aetiological groups on admission, the predictive value of these markers for the intracellular pathogen group was calculated. The area under the curve (AUC) of the ROC curves of low levels of YKL-40 or CCL18 for CAP caused by intracellular bacteria were 0.71 (95% CI 0.63–0.79) and 0.82 (95% CI 0.75–0.88), respectively. AUC curves of CCL18 and YKL-40 are shown in “Fig 3” next to AUC curve of CRP and procalcitonin.

The optimal cut-off limit for YKL-40 was calculated to be 140 ng/mL. AUC of YKL-40 levels below this cut-off value was 0.66 (95% CI 0.58–0.74). Optimal cut-off level for CCL18 was calculated to be 60 ng/mL, showing an AUC of 0.77 (95% CI 0.70–0.84) for levels below this cut-off value. For CRP, cut-off level was 200 mg/L with an AUC of 0.65 (95% CI 0.57–0.73). S4 Table shows percentages of patients per aetiological group above and below cut-off values for YKL-40 and CCL18, and the median values of the control group. YKL-40 level on admission of patients with CAP caused by an atypical pathogen was below the median value of the healthy controls in 13% of the patients; for CCL18 this was seen in 50% of the patients.

In multivariate analysis, both low levels of YKL-40 and CCL18 and high CRP on admission were significant predictors for CAP caused by an atypical pathogen. Low white blood cell count and age were predictors for CAP caused by an intracellular bacteria as well. Table 3 shows univariate and multivariate ORs with 95% CIs.

**Table 3:** Predictive value of YKL-40 and CCL18 for pneumonia caused by intracellular bacteria using univariate and multivariate logistic regression analysis.

	Univariate analysis		Multivariate analysis		Multivariate analysis	
	OR (95% CI)	*p*-value	OR (95% CI)	*p*-value	OR (95% CI)	*p*-value
YKL-40 <140 ng/mL	3.97 (2.09–7.53)	<0.001	3.08 (1.20–7.91)	0.019		
CCL18 <60 ng/mL	11.40 (5.53–23.52)	<0.001			9.11 (3.44–24.11)	<0.001
CRP >200 mg/L	3.70 (1.89–7.25)	<0.001	12.16 (4.62–31.99)	<0.001	11.85 (4.25–33.05)	<0.001
Age	0.97 (0.95–0.98)	<0.001	0.97 (0.95–0.99)	0.013	0.97 (0.95–1.00)	0.024
Male gender	1.80 (0.97–3.33)	0.064	1.95 (0.84–4.54)	0.120	2.27 (0.92–5.62)	0.076
Season—Spring	Reference value	0.008	Reference value	0.277	Reference value	0.164
Season–Summer	0.40 (0.14–1.12)	0.080	0.40 (0.10–1.59)	0.192	0.36 (0.08–1.56)	0.172
Season–Fall	0.64 (0.29–1.39)	0.258	1.16 (0.41–3.26)	0.778	2.11 (0.65–6.81)	0.213
Season–Winter	0.25 (0.11–0.58)	0.001	0.48 (0.17–1.35)	0.164	0.73 (0.24–2.22)	0.580
WBC count	0.84 (0.79–0.90)	<0.001	0.78 (0.71–0.86)	<0.001	0.79 (0.72–0.88)	<0.001
Procalcitonin	0.99 (0.96–1.02)	0.531	0.97 (0.94–1.01)	0.096	0.97 (0.94–1.01)	0.922

Abbreviations: CRP, C-reactive protein; WBC, white blood cell.

## Discussion

In 291 patients hospitalised with CAP, we studied the course of four pulmonary markers which have been extensively investigated in chronic pulmonary diseases. Their course and value in acute pulmonary infections is largely unknown.

We showed not only that YKL-40 and CCL18 levels were significantly higher at admission compared to levels of healthy controls, but, remarkably, both markers were still elevated on day 30. This finding indicates ongoing cellular activity 30 days after start of the pulmonary infection, a time point wherein most patients are expected to be in complete clinical remission.

Furthermore, we found higher YKL-40, CCL18 and SP-D levels during admission in patients with higher PSI scores (classes 4–5) while CRP and IL-6 levels did not significantly differ. Noteworthy, we found that both YKL-40 and CCL18 levels were lower on admission in CAP caused by an atypical pathogen compared to CAP caused by other or unknown aetiology. Dexamethasone treatment only influenced CCL-18 and SP-D course.

In patients hospitalised with CAP, Nordenbaek et al. found peaking YKL-40 levels one day after admission, that normalised after two days.[18] In our study, YKL-40 levels were highest on day 0, subsequently declined, but still remained elevated on day 30. Differences between our results and the results of Nordenbaek et al. may be caused by differences in severity between the CAP cohorts. We observed that YKL-40 remained elevated mainly in patients with higher PSI classes. Although Nordenbaek et al. did not calculate PSI scores, they did indicate that patients infected with *S*. *pneumoniae* were more seriously ill and had more widespread infiltrates on chest radiographs. Since we measured markers on days 0 and 2, we cannot rule out we missed a peaking level on day 1. Although, in 36 patients of whom we used serum of day 1 instead of serum of day 2 for marker measurement, no day 1 peak levels were observed either.

In accordance with our study, Wang et al. showed higher YKL-40 levels in patients with severe CAP.[28] We described YKL-40 to be a good predictor for length of hospital stay and CAP mortality.[13] The correlation of high pulmonary marker levels with severity of pneumonia could be explained by the extent of inflammation and subsequent repair of pulmonary damage, which might explain the higher degree of pulmonary marker release to the blood in case of severe pneumonia. Our finding that dexamethasone treatment, as part of the clinical trial participation, did not influence YKL-40 levels suggests it mainly is associated with tissue remodelling after CAP, rather than pro-inflammatory functions. SP-D as a marker for severity of pneumonia also has been studied before.[29–31] In accordance with the study of Leth-Larsen et al., we found SP-D levels to be significantly lower on day 0 in CAP patients compared to levels of healthy controls.[29] In contrast, two other studies showed high SP-D levels in respectively critically ill patients with acute respiratory distress syndrome (ARDS) suffering from A/H1N1 infection and CAP patients.[31,32] This difference in findings could be caused by the moment of sampling and/or that the study of Delgado et al. was performed in patients with ARDS, while our study excluded patient who were directly admitted to the intensive care unit (ICU). Lower SP-D levels found on day 0 might be due to less SP-D production by pneumocytes as a result of pulmonary infiltrates. SP-D levels were lowest in patients with viral pneumonia, possibly due to binding of SP-D with the virus to inhibit viral replication.[33] YKL-40 and CCL18 levels of viral CAPs were pretty similar to levels of CAPs with extracellular aetiology. Since there were only 19 patients in the viral CAP group and viral aetiology was heterogeneous speculations are difficult. One hypothesis is that viral infections can spread faster compared to bacterial infections, with the potential to cause more damage to epithelial cells in a short time. Since YKL-40 has a role in tissue remodelling after an inflammatory response, this could lead to higher YKL-40 levels. Another hypothesis is that our viral group was more severely ill compared to regular viral CAPs; CRP levels and PSI scores were relatively high for viral CAPs. Possibly bacterial superinfections were unidentified. Another possible explanation is the fact that the viral group contained (significantly) older patients and (although not significantly) more patients with chronic renal insufficiency compared to the other CAP groups.

Notably, we found levels of YKL-40 and CCL18 to be lower in patients with CAP caused by an intracellular, atypical bacteria as compared to patients with extracellular bacteria, for example *S*. *pneumoniae*. Levels of both markers were highest in case an extracellular bacteria was detected. This is in accordance with the study of Nordenbaek et al., which reported higher YKL-40 levels in patients with CAP caused by *S*. *pneumoniae*.[18] These higher levels indicate that *S*. *pneumoniae* causes more severe pneumonias compared to other pathogens. In our study, we chose to categorise aetiology in four groups to minimise number of levels in mixed model analysis. S3 Fig shows pulmonary marker levels for the ten main aetiologies, including *S*. *pneumoniae*.

Fifty percent of patients with CAP caused by an intracellular bacterium had suppressed CCL18 levels on admission since levels were below the median value of healthy controls. For YKL-40 this percentage was 13% (see S4 Table). This might be caused by consumption of CCL18 and YKL-40, comparable to the temporary decreased complement levels during an acute inflammatory response when demand shortly exceeds supply.[34] On admission, in atypical CAPs, IL-6 and leukocyte levels were lower compared with other pathogen while CRP levels were not. Considering the lower half-life of IL-6 and its early role in the inflammatory cascade compared to CRP, lower levels of leukocytes, CCL18 and YKL-40 on admission sketches a picture of a burst-like inflammatory response in atypical CAPs compared with bacterial CAPs, somewhat similar to viral CAPs as mentioned earlier. Alternatively, the lower YKL-40 and CCL18 levels in atypical CAP could suggest the inflammatory response is less intense. Intracellular bacteria might limit continued activation of innate inflammatory pathways of which YKL-40 and CCL18 are products. This theory is supported by lower levels of leucocytes and TNF-α, IL-6, IL-8 and IL-10 in our cohort in CAP caused by intracellular bacteria compared to CAP caused by *S*. *pneumoniae*.[35,36] Another explanation might be that the group with intracellular bacteria was younger and length of stay was shorter compared to other aetiology, suggesting a milder course. However, PSI scores and CRP levels were not significantly lower. Until now, the only biomarker known to differentiate between pathogens (viral or bacterial[37], gram-negative or gram-positive[38]) is procalcitonin. In both our univariate and multivariate analysis, procalcitonin levels were unable to distinguish between intracellular bacteria and other aetiologies. Combination of low YKL-40 and or low CCL18 and or high CRP levels did not improve prognostic value (data not shown). Therefore, individual levels of YKL-40 and especially CCL18 might be used to guide choice of microbiologic testing. In case of CA 15–3, mean levels were lower in patients with CAP caused by an extracellular bacteria, compared to the unknown group, even on day 30. This might indicate that extracellular bacteria cause less pulmonary damage and or fibrosis, since CA 15–3 is used as marker for pulmonary fibrosis.[5]

Dexamethasone lowered levels of CCL18 and IL-6 on days 2 and 4. This fits with the (known) capacity of dexamethasone to down regulate macrophages and monocytes, the two major cell types that are responsible for CCL18 production. SP-D levels were slightly higher on days 2 and 4 in the dexamethasone group. This could be explained by the fact that corticosteroids stimulate the production of surfactant phospholipids by alveolar type II cells through the fibroblast-pneumocyte factor, and enhance the expression of surfactant-associated proteins.[39] Surprisingly, YKL-40 levels were not influenced by dexamethasone treatment. We expected YKL-40 levels to be either higher in the dexamethasone group due to an enhancement of neutrophils by dexamethasone, or lower due to downregulation of inflammation by dexamethasone. Possibly these two opposing effects neutralise each other. CA 15–3 showed very little variation over time, perhaps due to a long biological half-life.

This study has several strengths. First, it is performed in a large, well-defined CAP cohort with a high fraction of identified pathogens. Second, to the best of our knowledge, it is the first study that investigates the relation of these four pulmonary markers with microbial aetiology. Last, it describes the effect of dexamethasone on these markers, giving more insight in the pathophysiology and treatment of a CAP episode.

A limitation of the study is that not of all patients samples were available for marker analysis on all four time-points. We used linear mixed modelling to decrease influence of this limitation on our results. Furthermore, information on smoking status was not available of all patients and was therefore chosen not to include in our analysis. Last, patients with comorbidities such as neoplastic diseases and COPD, diseases able to influence the markers under investigation, were included in our analysis. For aetiology, one of the main findings of our study, comorbidity percentages did not differ significantly between the different groups (see Table 1).

### Conclusions

SP-D, YKL-40 and CCL18 levels were higher in patients with severe pneumonia and YKL-40 and CCL18 levels were lower in CAP caused by intracellular, atypical bacteria compared to levels in CAP with other aetiology. These findings warrant further research into the role of these markers in the clinical management of CAP.

## Supporting information

S1 File Materials and methods. (DOC)

S1 Table Baseline characteristics of patients in whom samples were or were not available on day 4 and day 30. * Indicates a significant difference between available and not available samples after Bonferroni correction for multiple testing, a *p*-value<0.0042; Abbreviations: CKD, chronic kidney disease; COPD, chronic obstructive pulmonary disease; n, number; PSI, pneumonia severity index.
(DOC)

S2 Table Available marker levels of 291 patients hospitalised with community-acquired pneumonia. (DOC)

S3 Table Overview of the variables included in the final linear mixed model analysis for each pulmonary marker. If a *p*-value is given the marker was included in the final model. * Time: Indicates an interaction with time.
(DOC)

S4 Table Number of patients per aetiology below the median values of the healthy controls and above the cut-off values of respectively YKL-40 and CCL18. (DOC)

S1 Fig Change in log normally-transformed pulmonary marker levels over time dependent on antibiotic use before hospitalisation. (DOC)

S2 Fig Change in log normally-transformed pulmonary marker levels over time dependent on dexamethasone use. (DOC)

S3 Fig Course of (A) YKL-40, (B) CCL18, (C) CA 15–3 and (D) SP-D in patients hospitalised with community-acquired pneumonia categorised according to aetiology.
(DOC)
