# Supplementary material for: Course of SP-D, YKL-40, CCL18 and CA 15-3 in adult patients hospitalised with community-acquired pneumonia and their association with disease severity and aetiology: A post-hoc analysis
Source: PLoS One. 2018 Jan 11;13(1):e0190575. doi: 10.1371/journal.pone.0190575 (PMC5764260; doi:10.1371/journal.pone.0190575)
Supplement: S1 File — (DOC) [file pone.0190575.s001.doc]

**Supporting information file 1**

*belonging to the manuscript entitled “Course of SP-D, YKL-40, CCL18 and CA 15-3 in adult patients hospitalised with community-acquired pneumonia and their association with disease severity and aetiology: a post-hoc analysis” by Spoorenberg et al.*

**MATERIALS AND METHODS**

**Microbiologic testing**

Blood cultures were taken and, if available, cultured and Gram stained. If available, sputum samples were gram stained, cultured, and analysed with TaqMan real-time polymerase chain reactions (PCRs) in order to detect DNA of Mycoplasma pneumoniae, Legionella species, Coxiella burnetii, and Chlamydophila species. Antigen testing of Streptococcus pneumoniae and Legionella pneumophila type 1 was performed in urine samples. Paired complement fixation test was performed for the presence of antibodies to Mycoplasma pneumoniae, Coxiella burnetii, Chlamydophila species and the viruses adenovirus, influenza virus A and B, parainfluenza virus 1, 2 and 3, and the respiratory syncytial virus. A fourfold increase in antibody titre was considered as positive. Furthermore, culture and PCR was performed on pharyngeal swabs to detect (para)influenza virus, adenovirus and respiratory syncytial virus.

If in one patient both a bacterium and virus were detected, the bacterial species was considered the causative pathogen. If two different bacterial species were found, the pathogen known to most likely cause CAP was considered causative.

**Measurement of pulmonary markers: quantification levels**

For YKL-40 and SP-D measurement, 20 μL of serum was used and diluted 10 times. The lower limit of quantification (LLOQ) of YKL-40 was 4.4 ng/mL and the upper limit of quantification (ULOQ) 2423.5 ng/mL. The LLOQ of SP-D was 1.73 ng/mL and the ULOQ 946.50 ng/mL. For CCL18 measurement and CA 15-3 10 μL was used and diluted 150 times. The LLOQ for CCL18 was 9.0 ng/mL and the ULOQ 1485.0 ng/mL. For CA 15-3 the LLOQ was 2.0 U/mL and the ULOQ 8100.0 U/mL.

**Data analyses: linear mixed modelling and ROC curve and regression analysis**

In mixed model analysis, variables that possibly influenced course of the pulmonary markers were used in model building. The primary explanatory parameters were 1) the repeated observations (hereafter called ‘time’), 2) PSI classes 4-5, and the interaction between the latter two. These three parameters constitute the basic model. Subsequently the following parameters were added to the model including their interaction with time: COPD-presence, antibiotic use before hospitalisation, dexamethasone use, and aetiology. The -2 log-likelihood (-2LL) is the fit measure of choice in linear mixed modelling: it is a unitless value and it serves the same goal as the correlation coefficient in linear regression. The difference with the latter is that this value should decrease as the fit between data and model becomes better. The value for an ‘extended’ model was compared to the basic one and when the -2LL significantly decreased (based on a χ^2^ test), the added parameter was retained. This better fitting model then formed the new ‘basic’ model to which new parameters were added. This cycle was repeated until we found the best fitting model.

ROC-curve analysis was used to evaluate the predictive value of a given pulmonary marker level on admission for atypical CAP aetiology, using atypical pathogen versus all other aetiology (bacterial, viral and unknown). The marker level combining the highest sensitivity and specificity was selected as threshold value. These threshold values were used in univariate and multivariate logistic regression analysis to analyse predictive value of these markers for CAP caused by an atypical pathogen. Variables known to be associated with CAP caused by an atypical pathogen were used (age, male gender, and season), and differences in baseline characteristics a *p*-value <0.10 were used in regression analyses.
