# Supplementary material for: Course of SP-D, YKL-40, CCL18 and CA 15-3 in adult patients hospitalised with community-acquired pneumonia and their association with disease severity and aetiology: A post-hoc analysis
Source: PLoS One. 2018 Jan 11;13(1):e0190575. doi: 10.1371/journal.pone.0190575 (PMC5764260; doi:10.1371/journal.pone.0190575)
Supplement: S1 Table — * Indicates a significant difference between available and not available samples after Bonferroni correction for multiple testing, a p-value<0.0042; Abbreviations: CKD, chronic kidney disease; COPD, chronic obstructive pulmonary disease; n, number; PSI, pneumonia severity index. (DOC) [file pone.0190575.s002.doc]

**S1 Table**

*belonging to the manuscript entitled “Course of SP-D, YKL-40, CCL18 and CA 15-3 in adult patients hospitalised with community-acquired pneumonia and their association with disease severity and aetiology: a post-hoc analysis” by Spoorenberg et al.*

**Baseline characteristics of patients in whom** samples were or were not available on day 4 and day 30.

|  | Day 4 available  (n=221) | Day 4  not available  (n=70) | Day 30  available  (n=209) | Day 30  not available  (n=82) |
| --- | --- | --- | --- | --- |
| Male sex (%) | 129 (58.4) | 34 (48.6) | 115 (55.0) | 48 (58.5) |
| Age (year) (±) | 65.4 (17.5) | 59.5 (20.7) | 60.8 (18.0) | 72.1 (17.0)* |
| Comorbidities (%)  CKD  Diabetes mellitus  Liver disease  Neoplastic disease  Heart failure  COPD | 24 (10.9)  34 (15.4)  0  15 (6.8)  38 (17.2)  24 (10.9) | 4 (5.7)  7 (10.0)  2 (2.9)  4 (5.7)  9 (12.9)  8 (11.4) | 17 (8.1)  32 (15.3)  1 (0.5)  13 (6.2)  27 (12.9)  21 (10.0) | 11 (13.4)  9 (11.0)  1 (1.2)  6 (7.3)  20 (24.4)  11 (13.4) |
| PSI classes (%)  Classes 1-3  Classes 4-5 | 113 (51.1)  108 (48.9) | 39 (55.7)  31 (44.3) | 123 (58.9)  86 (41.4) | 29 (35.4)*  53 (64.6)* |
| Pathogen (%) |  |  |  |  |
| Atypical | 38 (17.2) | 17 (24.3) | 46 (22.0) | 9 (11.0)* |
| Other bacteria  Virus or unknown | 74 (33.5)  109 (49.3) | 16 (22.9)  37 (52.9) | 77 (36.8)  86 (41.1) | 13 (15.9)*  60 (73.2)* |
| Dexamethasone (%)^‡^ | 110 (49.8) | 35 (50) | 104 (49.8) | 41 (50.0) |

* Indicates a significant difference between available and not available samples after Bonferroni correction for multiple testing, a *p*-value<0.0042; Abbreviations: CKD, chronic kidney disease; COPD, chronic obstructive pulmonary disease; n, number; PSI, pneumonia severity index.
