# Supplementary material for: Course of SP-D, YKL-40, CCL18 and CA 15-3 in adult patients hospitalised with community-acquired pneumonia and their association with disease severity and aetiology: A post-hoc analysis
Source: PLoS One. 2018 Jan 11;13(1):e0190575. doi: 10.1371/journal.pone.0190575 (PMC5764260; doi:10.1371/journal.pone.0190575)
Supplement: S2 Table — (DOC) [file pone.0190575.s003.doc]

**S2 Table**

*belonging to the manuscript entitled “Course of SP-D, YKL-40, CCL18 and CA 15-3 in adult patients hospitalised with community-acquired pneumonia and their association with disease severity and aetiology: a post-hoc analysis” by Spoorenberg et al.*

Available marker levels of 291 patients hospitalised with community-acquired pneumonia.

|  | Pulmonary markers | C-reactive protein | Interleukin-6 |
| --- | --- | --- | --- |
| Day 0 | 289 (99.3%) | 291 (100%) | 280 (96.2%) |
| Day 2 | 277 (95.2%) | 279 (95.9%) | 194 (66.7%) |
| Day 4 | 221 (75.9%) | 253 (86.9%) | 158 (54.3%) |
| Day 30 | 209 (71.8%) | 219 (75.3%) | 78 (26.8%) |
| Total | 291 (100%) | 291 (100%) | 291 (100%) |
