# Supplementary material for: Course of SP-D, YKL-40, CCL18 and CA 15-3 in adult patients hospitalised with community-acquired pneumonia and their association with disease severity and aetiology: A post-hoc analysis
Source: PLoS One. 2018 Jan 11;13(1):e0190575. doi: 10.1371/journal.pone.0190575 (PMC5764260; doi:10.1371/journal.pone.0190575)
Supplement: S3 Table — If a p-value is given the marker was included in the final model. * Time: Indicates an interaction with time. (DOC) [file pone.0190575.s004.doc]

**S3 Table**

*belonging to the manuscript entitled “Course of SP-D, YKL-40, CCL18 and CA 15-3 in adult patients hospitalised with community-acquired pneumonia and their association with disease severity and aetiology: a post-hoc analysis” by Spoorenberg et al.*

Overview of the variables included in the final linear mixed model analysis for each pulmonary marker.

|  | YKL-40 | CCL18 | CA 15-3 | SP-D | CRP | Interleukin-6 |
| --- | --- | --- | --- | --- | --- | --- |
| Time | <0.001 | 0.358 | <0.001 | <0.001 | <0.001 | <0.001 |
| PSI severity | <0.001 | <0.001 | 0.179 | 0.001 | 0.036 |  |
| PSI severity * Time |  | 0.017 | 0.038 | 0.014 | <0.001 |  |
| Aetiology | <0.001 | <0.001 | 0.005 | 0.055 | <0.001 | 0.097 |
| Aetiology * Time | <0.001 | <0.001 |  | 0.004 | 0.002 | 0.001 |
| Antibiotic use | 0.030 |  | 0.018 |  |  | 0.062 |
| Antibiotic use * Time |  |  |  |  |  | 0.026 |
| Dexamethasone |  | <0.001 |  | 0.788 | 0.027 | 0.055 |
| Dexamethasone * Time |  | <0.001 |  | 0.023 | <0.001 | <0.001 |

If a *p*-value is given the marker was included in the final model. * Time: Indicates an interaction with time.
