# Supplementary material for: Course of SP-D, YKL-40, CCL18 and CA 15-3 in adult patients hospitalised with community-acquired pneumonia and their association with disease severity and aetiology: A post-hoc analysis
Source: PLoS One. 2018 Jan 11;13(1):e0190575. doi: 10.1371/journal.pone.0190575 (PMC5764260; doi:10.1371/journal.pone.0190575)
Supplement: S4 Table — (DOC) [file pone.0190575.s005.doc]

**S4 Table**

*belonging to the manuscript entitled “Course of SP-D, YKL-40, CCL18 and CA 15-3 in adult patients hospitalised with community-acquired pneumonia and their association with disease severity and aetiology: a post-hoc analysis” by Spoorenberg et al.*

Number of patients per aetiology below the median values of the healthy controls and above the cut-off values of respectively YKL-40 and CCL18.

|  |  | Atypical bacteria  n:54 | Extracellular bacteria  n:90 | Viruses  n:19 | Unknown aetiology  n:126 |
| --- | --- | --- | --- | --- | --- |
| YKL-40 in ng/mL | <23 (median of controls) (%) | 7 (13) | 3 (3) | 1 (5) | 4 (3) |
|  | 23-140 (%) | 31 (57) | 24 (27) | 4 (21) | 52 (41) |
|  | >140 (cut-off value) (%) | 16 (30) | 63 (70) | 14 (74) | 70 (56) |
| CCL18 in ng/mL | <38 (median of controls) (%) | 27 (50) | 7 (8) | 5 (26) | 16 (13) |
|  | 38-60 (%) | 16 (30) | 9 (10) | 0 | 23 (18) |
|  | >60 (cut-off value) (%) | 11 (20) | 74 (82) | 14 (74) | 87 (69) |
