# Supplementary material for: Course of SP-D, YKL-40, CCL18 and CA 15-3 in adult patients hospitalised with community-acquired pneumonia and their association with disease severity and aetiology: A post-hoc analysis
Source: PLoS One. 2018 Jan 11;13(1):e0190575. doi: 10.1371/journal.pone.0190575 (PMC5764260; doi:10.1371/journal.pone.0190575)

**S1 Fig**

*belonging to the manuscript entitled “Course of SP-D, YKL-40, CCL18 and CA 15-3 in adult patients hospitalised with community-acquired pneumonia and their association with disease severity and aetiology: a post-hoc analysis” by Spoorenberg et al.*

**Change in log normally-transformed pulmonary marker levels over time dependent on antibiotic use before hospitalisation.**


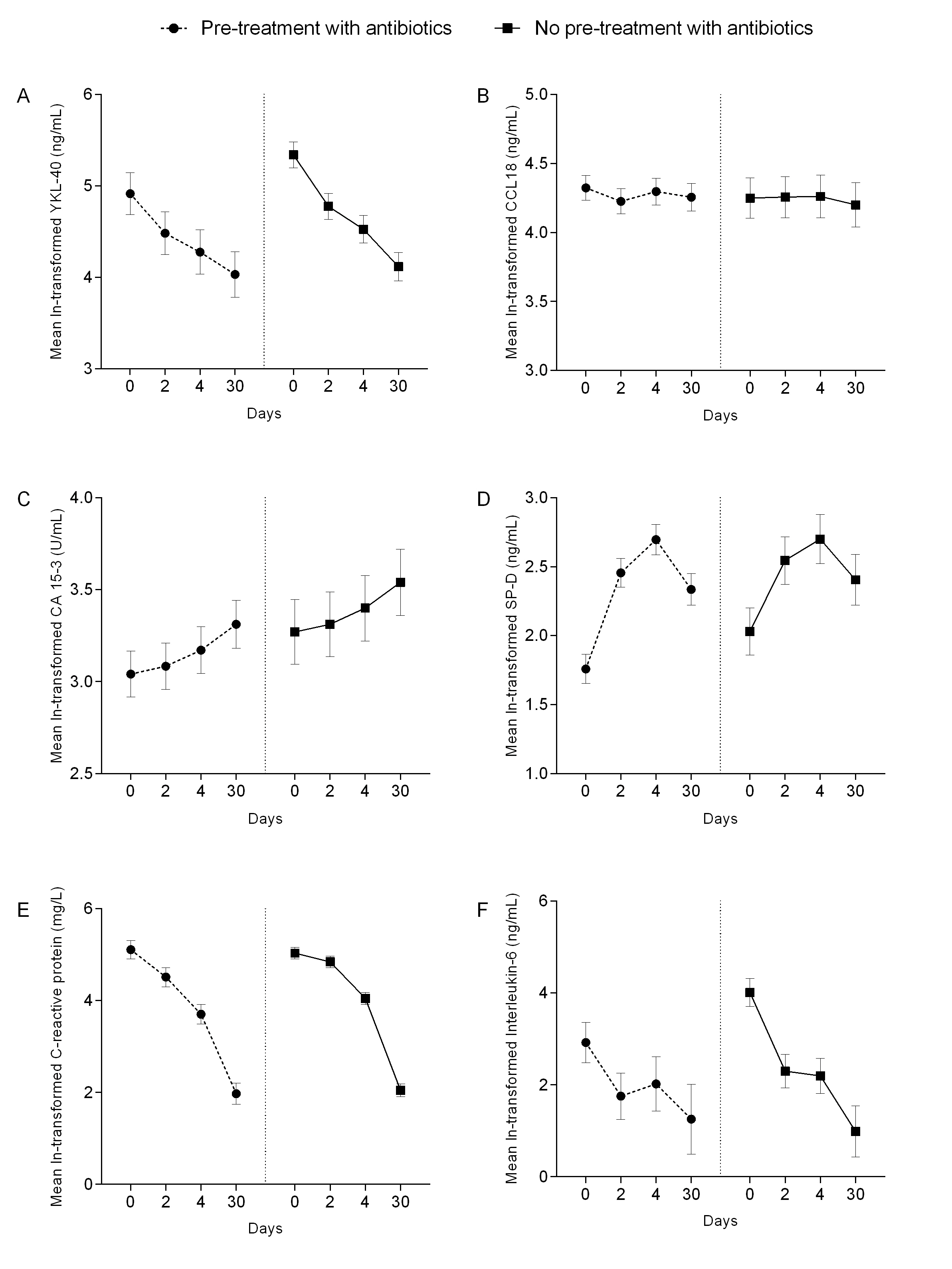

Supplement: S1 Fig — (DOC) [file pone.0190575.s006.doc]
