# Supplementary material for: Course of SP-D, YKL-40, CCL18 and CA 15-3 in adult patients hospitalised with community-acquired pneumonia and their association with disease severity and aetiology: A post-hoc analysis
Source: PLoS One. 2018 Jan 11;13(1):e0190575. doi: 10.1371/journal.pone.0190575 (PMC5764260; doi:10.1371/journal.pone.0190575)
Supplement: S3 Fig — Course of (A) YKL-40, (B) CCL18, (C) CA 15–3 and (D) SP-D in patients hospitalised with community-acquired pneumonia categorised according to aetiology. (DOC) [file pone.0190575.s008.doc]

**S3 Fig**

*belonging to the manuscript entitled “Course of SP-D, YKL-40, CCL18 and CA 15-3 in adult patients hospitalised with community-acquired pneumonia and their association with disease severity and aetiology: a post-hoc analysis” by Spoorenberg et al.*

**Course of (A) YKL-40, (B) CCL18, (C) CA 15-3 and (D) SP-D in patients hospitalised with community-acquired pneumonia categorised according to aetiology.**


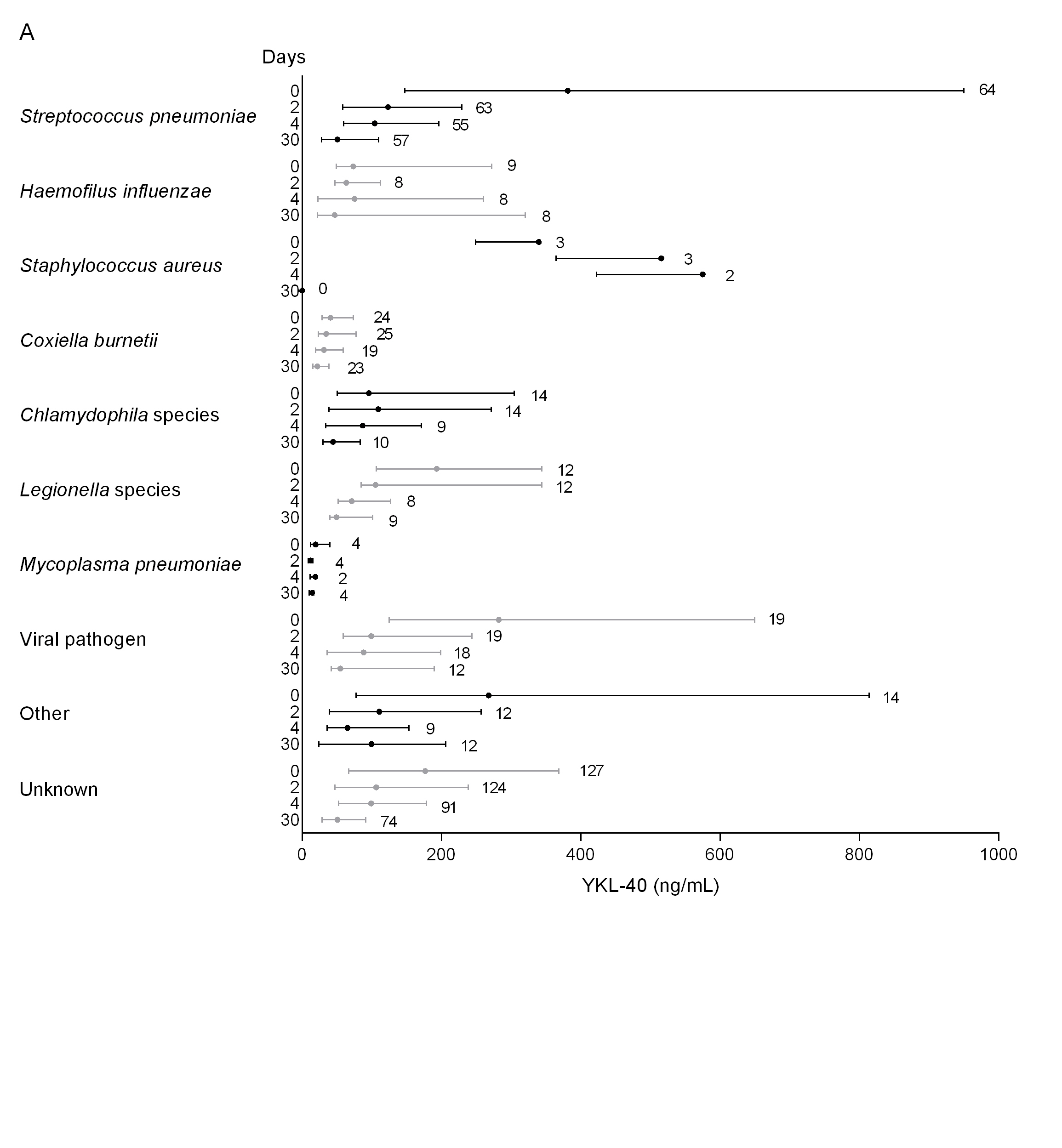


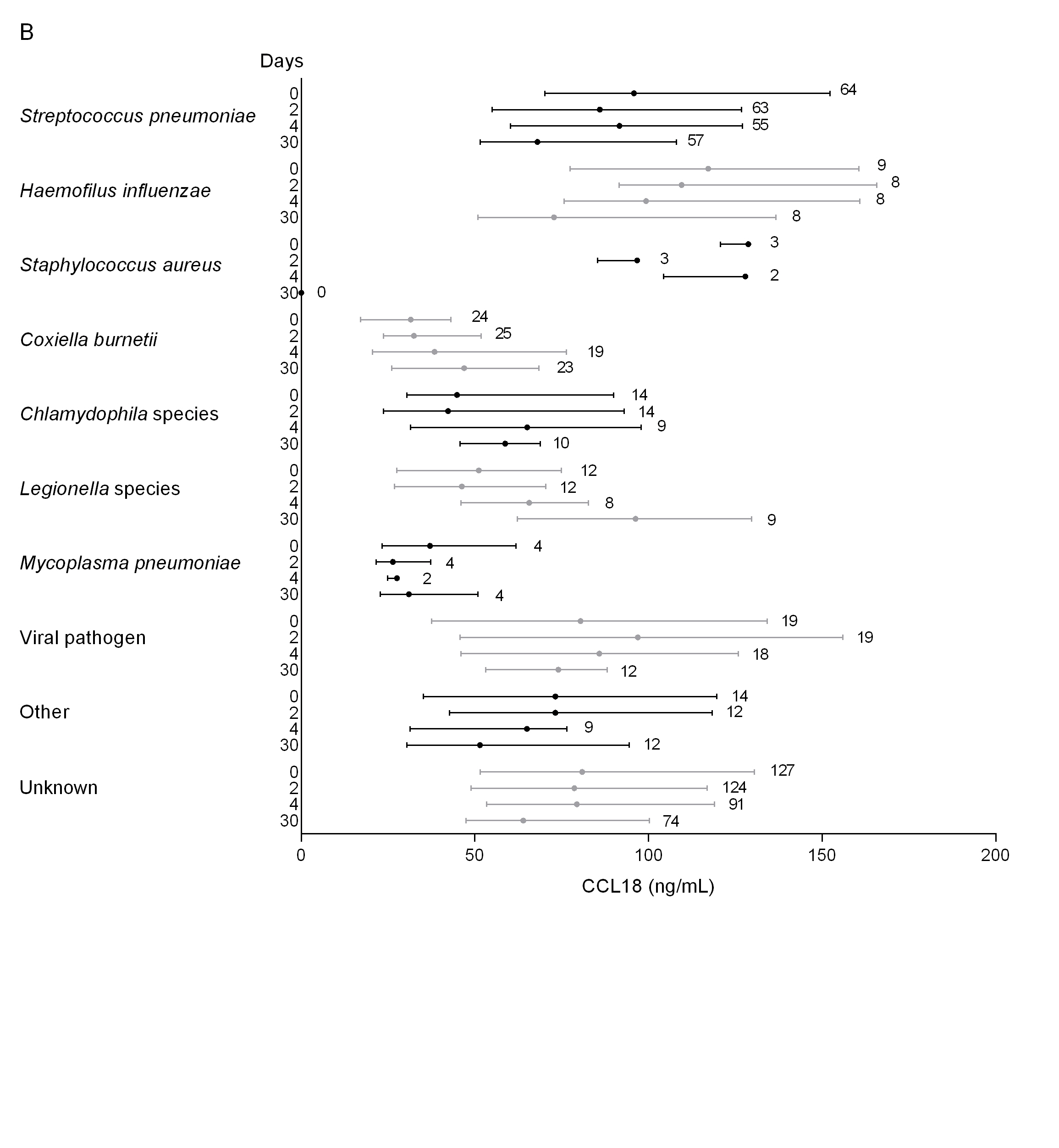


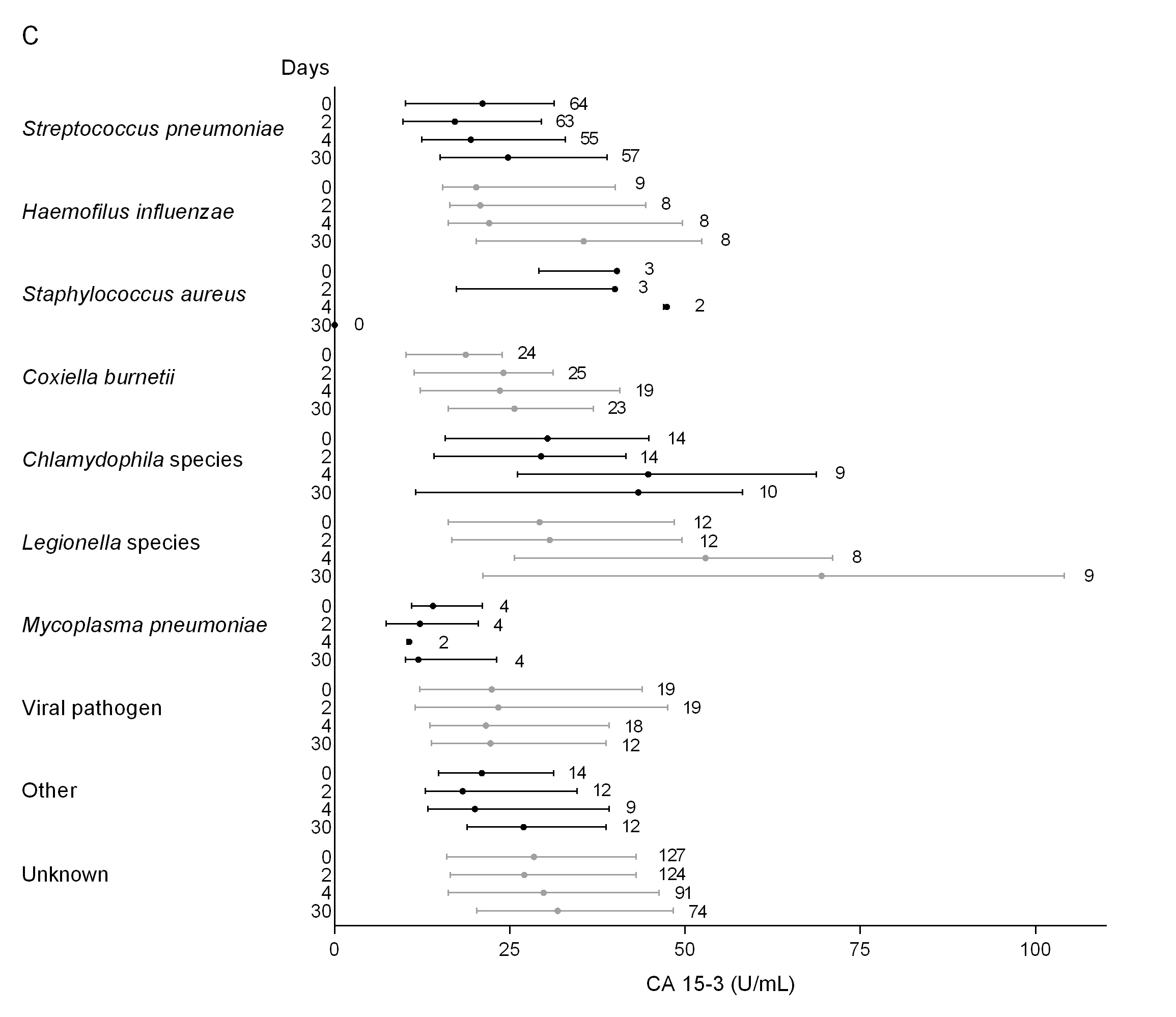


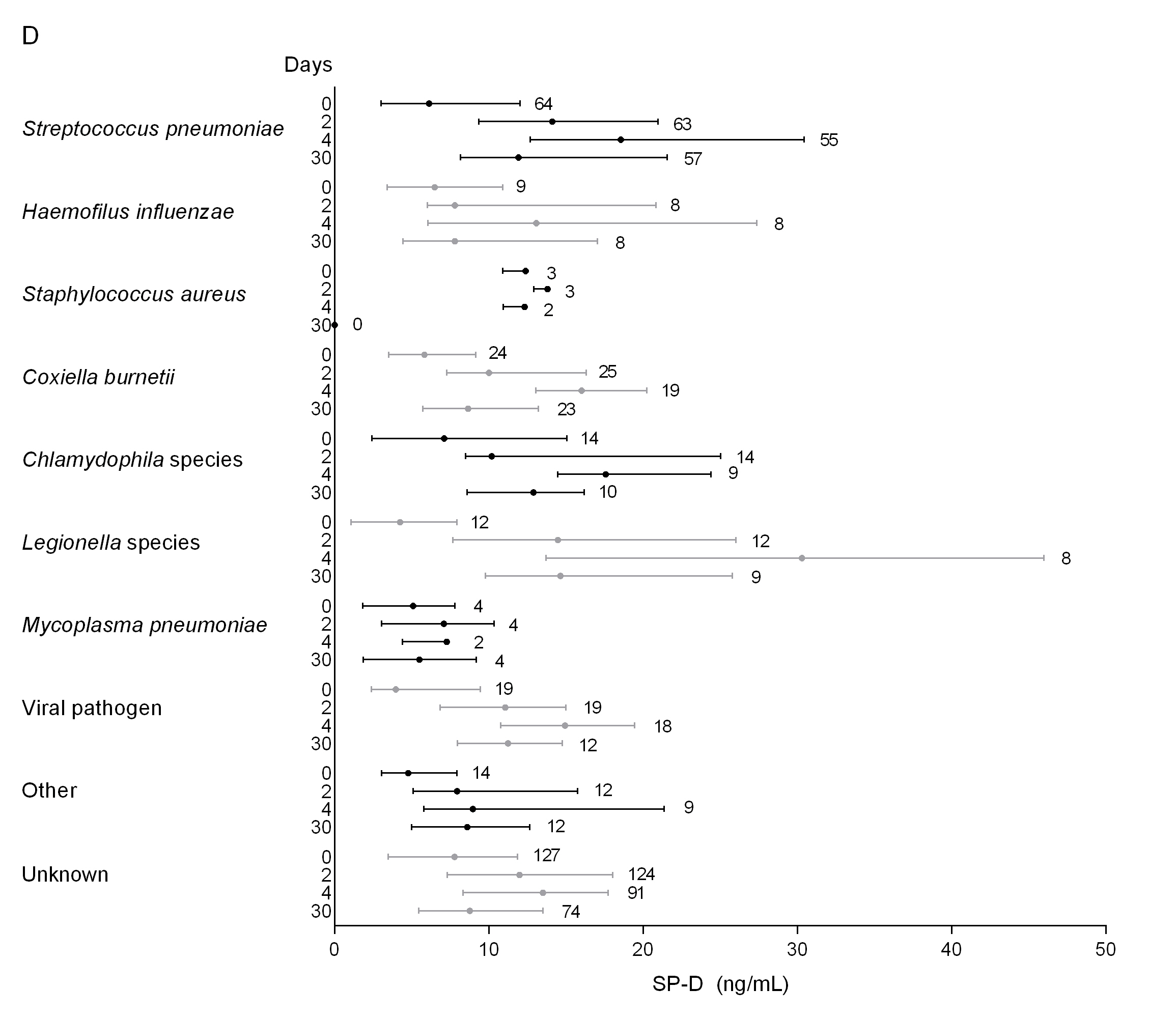


Levels are indicated in median with interquartile ranges. The numbers indicate the number of patients. The group ‘other’ comprised of 4x gram-negative rods, 2x *Escherichia coli*, 2x *Haemophilus parainfluenzae*, 2x *Streptococcus group A*, 1x *Pseudomonas aeruginosa*, 1x *Klebsiella pneumoniae*, 1x *Rhodococcus equi* and 1x *Streptococcus* group G.
